# Plasma Concentrations of Cytokines in Patients with Combined Hyperlipidemia and Atherosclerotic Plaque before Treatment Initiation—A Pilot Study

**DOI:** 10.3390/medicina58050624

**Published:** 2022-04-29

**Authors:** Marcin Basiak, Michal Kosowski, Marcin Hachula, Boguslaw Okopien

**Affiliations:** Department of Internal Medicine and Clinical Pharmacology, Medical University of Silesia, Medykow 18, 40-752 Katowice, Poland; mkosowski@sum.edu.pl (M.K.); mhachula@sum.edu.pl (M.H.); bokopien@sum.edu.pl (B.O.)

**Keywords:** atherosclerosis, hyperlipidemia, rupture plaque, interleukin 6, interleukin 18, tumor necrosis factor alfa

## Abstract

*Background and Objectives*: The formation and destabilization of atherosclerotic plaques is a complex process involving several proteins and cytokines. Interleukin 6 (IL-6), interleukin 18 (IL-18), and tumor necrosis factor (TNF-α) are examples of such cytokines. The goal of our research is to compare the concentrations of the above-mentioned indicators in the plasma of patients with verified high-risk atherosclerotic plaque to the plasma levels of healthy people before lipid lowering therapy. *Materials and Methods*: Patients with dyslipidemia who had the presence of unstable atherosclerotic plaque verified by ultrasonography were included in the study. The concentrations of IL-6, IL-18 and TNF-α in the plasma of these people were determined and compared with the concentrations of these cytokines in the plasma of the control group. *Results*: Levels of lipid panel, IL-6 and IL-18 were significantly lower in the group of healthy people than in the study group. *Conclusions*: The concentrations of IL-6 and IL-18 in the plasma of patients with ruptured plaque are higher than in the plasma of healthy people, suggesting that these cytokines as a panel might be used as new indicators of the presence of unstable atherosclerotic plaque.

## 1. Introduction

Complications of atherosclerosis are the leading causes of morbidity and mortality worldwide, accounting for over 19 million deaths annually [1]. The hypothesis of atherosclerosis development treats it as a chronic inflammatory vascular wall response initiated by endothelial damage. The process of atherosclerotic plaque formation is triggered by an ongoing inflammatory process and involves both endothelial dysfunction and high levels of circulating cholesterol. In the sub-epithelial space of the endothelium damaged by the inflammatory process, foam cells accumulate, thus creating an atherosclerotic plaque, which, as it increases in size, may result in complete occlusion of the artery involved in the atherosclerotic process [2]. Moreover, it enhances the translocation of transcriptional factors for pro-inflammatory cytokine genes into the cell nucleus and reduces the formation of anti-inflammatory cytokines in macrophages [3,4]. The genesis and progression of atherosclerotic lesions is accompanied by a cascade of secretions of inflammatory markers and an increase in chemotactic activity. That inflammatory processes play a crucial role in atherogenesis is reflected by the presence of large numbers of inflammatory cells, mainly monocytes/macrophages and T lymphocytes, within the atherosclerotic plaque [2,5]. The most precise predictors of atherosclerotic plaque destabilization are imaging methods such as ultrasound and the much more sensitive and specific CT and MRI [6]. However, these are more costly and less practical methods than trying to determine and describe plasma indices of plaque destabilization. High-risk or unstable plaques are associated with an increased risk of rupture, uncontrolled embolization, and vascular incidents. Changes in the histological structure of such plaque show a large lipid core, a thin cover covered with fibrin, and may show ulceration, the presence of parietal thrombi or effusions into the plaque, as well as intense infiltration of macrophages and other inflammatory cells [7]. Activated macrophages, T cells, and mast cells produce pro-inflammatory cytokines, proteinases, coagulation factors, and adhesive molecules that may be responsible for the progression and destabilization of atherosclerotic lesions [8,9,10].

Cytokines are key regulatory glycoproteins associated with inflammatory and immune processes that modulate all aspects of vascular inflammation. Many cytokines are involved in the onset and complications of atherosclerosis. For our analysis, we chose three cytokines that lead in the processes of atherosclerosis: interleukin 6 (IL-6), interleukin 18 (IL-18) and tumor necrosis factor α (TNF-α).

The role of (Il-6) in the development of atherosclerosis has been widely described in InCHIANTI and MESA studies as an independent predictor of peripheral arterial disease (PAD) in community screening, irrespective of ethnicity. Il-6 is an interleukin produced in our bodies by many types of cells, such as macrophages, monocytes, endothelium cells and adipose tissue cells. In monocytes and macrophages especially, its expression increases in response to factors such as viruses, bacterial toxins or the inflammatory process caused by other interleukins such as, for example, TNF-α [11,12]. The IL-6 produced by these cells affects the vascular endothelial cells, leading to an increase in the concentration of adhesion molecules and chemoattractants, which increases the influx of neutrophils into this tissue [13,14]. Moreover, Il-6 causes the overproduction and reactivity of acute phase markers such as C-reactive protein (CRP) and TNF-α [15,16]. Additionally, IL-6 takes part in smooth muscle cells (SMCs) migration by influence on vascular endothelial growth factor (VEGF) and TNF-α [17,18].

IL-18 has a crucial role in the progression and stabilization of atherosclerotic plaque [19].

In experimental studies, the administration of exogenous IL-18 to mice caused an accelerated development of atherosclerotic plaques [20].

TNF-α is present at every stage of the progression of atherosclerosis. It stimulates the expression of adhesion molecules, selectin and the production of metalloproteinases in the vascular endothelium [21]. Locally, within the atherosclerotic plaque, it increases the expression of tissue factor, a potent thrombogenic protein [22].

It is important to find new soluble markers that can assess the risk of atherosclerotic plaque rupture in patients with dyslipidemia.

In our study, we assessed the concentrations of the above-mentioned markers in the serum of patients suffering from mixed hyperlipidemia with a high risk of atherosclerotic plaque and compared them with the population of healthy subjects.

## 2. Materials and Methods

Patients were recruited for the study from persons 18–75 years of age, screened in our department for the presence of asymptomatic atherosclerosis by the sonographic assessment of common carotid intima-media thickness.

Subjects were eligible for the study if they met the following criteria: mixed hyperlipidemia (former Frederickson hyperlipidemia type 2B)-plasma total cholesterol (TC) > 200 mg/L low-density lipoprotein cholesterol (LDL) > 135 mg/dL, triglycerides (TG) > 150 mg/dL; for women at least 24 months since the last menstruation, hysterectomy or ovarectomy, or using mechanical contraception.

The exclusion criteria were as follows: other types of primary dyslipidemias; secondary dyslipidemia in the course of autoimmune disorders, thyroid diseases, chronic pancreatitis, nephrotic syndrome, liver and biliary tract diseases, obesity (body mass index (BMI) > 30 kg/m^2^) or alcoholism; any acute and chronic inflammatory processes; symptomatic congestive heart failure; unstable coronary artery disease, myocardial infarction or stroke within 6 months preceding the study; arterial hypertension; impaired renal or hepatic function; malabsorption syndromes; treatment with other hypolipidemic drugs within 3 months before the study; taking drugs interfering with studied substances’ serum levels or affecting lipid metabolism (e.g., statins, fibrates, ezetymibe, niacin, non-selective beta-blockers); concomitant treatment with drugs that may affect inflammatory processes in the vascular wall (including nonsteroid anti-inflammatory drugs and angiotensin converting enzyme inhibitors) within 3 months before the enrollment; ongoing hormonal replacement therapy or oral contraception.

Specific inclusion criteria for high risk atherosclerotic plaque: Ultrasonography is a method of atherosclerotic carotid disease evaluation; it is clinically used to assess the presence of plaque, the degree of carotid stenosis with blood-flow velocity profiles, and the carotid intima-media thickness. The examination of the carotid arteries and assessment of complex intima media thickness (C-IMT) in the extracranial segment was performed using B-mode ultrasound with a linear probe at a frequency of 7, 5–10 MHz. The C-IMT was evaluated three times, and the mean score was taken into consideration. The measure was performed in the distal common carotid (1 cm proximal to the carotid bulb). Unstable plaques were associated with fibrofatty and hemorrhagic content, with an echolucent appearance, as previously described [23]. In addition, high risk plaques may have an irregular surface or ulcerations, which were detected with the Color–Doppler method [24].

Of these 128 screened patients, 18 fulfilled all the very detailed and narrow inclusion criteria and were eligible for study entry.

All patients gave their written informed consent in accordance with the Declaration of Helsinki. The study protocol was approved by the Bioethical Committee of the Medical University of Silesia. Because lipid-lowering therapy is of proven value, the patients were then treated with high-dose lipid-lowering therapy.

Therefore, our control group included 12 age-, sex- and weight-matched healthy subjects.

Taking history, clinical examination and venous blood sampling for evaluating safety laboratory parameters were performed before the start of therapy. Laboratory tests included total and differential blood cell count, blood sedimentation rate, creatine kinase, alanine and aspartate aminotransferases, gamma-glutamyltransferase, alkaline phosphatase, electrolytes, bilirubin, creatinine, total proteins, urine examination, glycated hemoglobin and 12-lead electrocardiography (ECG).

Venous blood samples were collected after an overnight 12 h fasting at 8 a.m. before the treatment. All the tests were carried out by a person blinded to the subject’s identity and all clinical details. Plasma lipids and glucose were assayed by routine laboratory techniques and LDL cholesterol levels were measured directly. The plasma levels of IL-6, IL-18 and TNF-α were assessed by commercially available enzyme immunoassay methods using Cloud-Clone Corp. USA and Diaclone, France kits as described by the manufacturer. All the laboratory tests were also performed in the control group.

### Statistical Analysis

The collected data were processed via the Statistica TIBCO Software Inc. Palo Alto, Santa Clara, CA, USA (2017) version 13.3 program, licensed by the Medical University of Silesia in Katowice. The Shapiro–Wilk test was used to assess the normality of distributions. To compare quantitative variables, the *t*-test for independent means or the U Mann–Whitney test (in the case of non-compliance with the conditions of the *t*-test) was used. We also used Spearman’s rank correlation to assess the relationship between variables. We assumed a *p*-value of less than 0.05 as statistically significant.

## 3. Results

Study groups did not differ significantly in terms of demographic data (age, gender, smoking and weight). After reviewing patient records, it was discovered that patients in both groups had not received any medications in the three months preceding enrollment.

In the control group, statistically significantly lower concentrations of TC, LDL, non-high-density lipoprotein cholesterol (non-HDL) and TG (*p* < 0.001) were observed compared to the study group. However, no statistically significant differences in high-density lipoprotein cholesterol (HDL) levels were observed (*p* = 0.47). (Table 1).

We have also verified the relationship between the concentrations of interleukins and individual cholesterol fractions, proving the presence of a positive correlation between the concentrations of Il-18 and Il-6, and TC, LDL, TG and non-HDL. Such a correlation was not observed between interleukins concentrations and HDL levels. Detailed results are presented in Table 2.

Moreover, in the control group, the levels of Il-18 and Il-6 were also statistically significantly lower than in the study group (*p* < 0.05). There were no statistically significant differences between groups with regard to TNFα concentration (*p* = 0.92).

**Figure 1:**
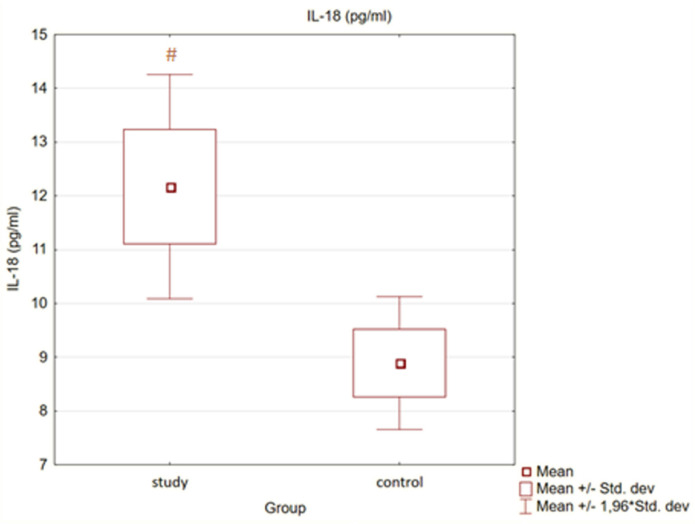
Concentration of interleukin 18 (Il-18) in study and control group (#—*p* < 0.05 versus control).

**Figure 2:**
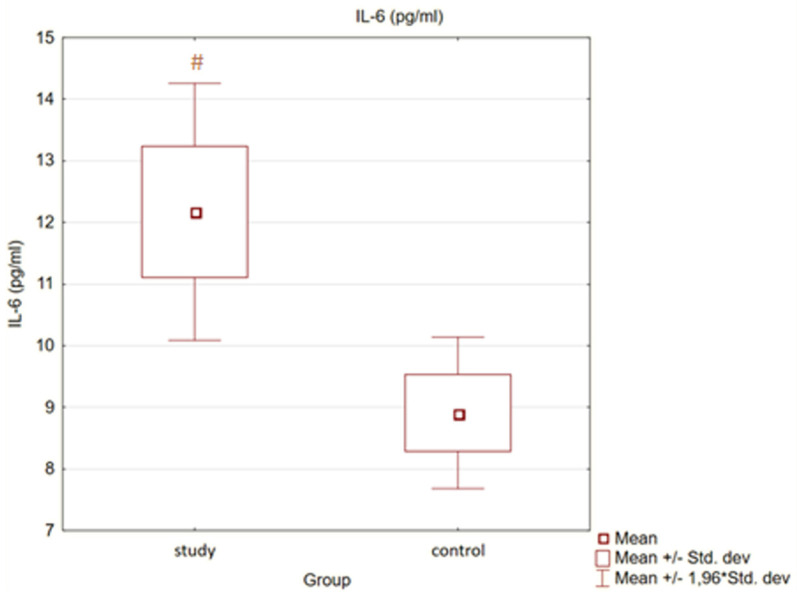
Concentration of interleukin 6 (Il-6) in study and control group (#—*p* < 0.05 versus control).

Detailed results are presented in Figure 1 and Figure 2.

## 4. Discussion

Atherosclerosis and its complications in the form of major cardiovascular incidents are an increasingly frequent cause of death [25]. It is the reason why the investigation of such biochemical parameters was started, which will enable even better estimation of the risk of a cardiovascular event in patients compared to the lipid profile determinations used so far. The researchers’ attention is particularly focused on cytokines and proteins that may be responsible for the stabilization of atherosclerotic plaque, and thus influence this risk.

In our study, we proved that in the group of people suffering from lipid disorders, in whom the presence of high risk plaque was confirmed by ultrasound methods, we could observe different concentrations of cytokines, such as IL-6, IL-18, compared to the group of healthy people, which are involved in the formation and stabilization of atherosclerotic plaque. Moreover, we proved the correlation between the concentration of interleukins and serum lipid levels. So far, only a single study has shown a relationship between the concentration of cholesterol fraction and the concentrations of pro-inflammatory interleukins. In a study conducted on a group of obese South African women, the existence of a relationship between the concentration of interleukin-18 and TG and HDL was proven, the results of which are consistent in terms of TG level with those observed in our group of patients [26]. Toader MP et al. also proved that the concentration of IL-6 is related to the levels of TC and HDL. However, no correlation between IL-6 and other cholesterol fractions was noticed in this study [27]. An inverse relationship was observed by Kim S.J. et al. in their study, in which they described a negative correlation between the concentration of anti-inflammatory interleukin 10 and TC and LDL [28].

Despite numerous studies of lipid disorders’ sequels, the exact mechanisms triggering and enhancing the remodeling of the arterial wall are still not fully recognized. The key factor in the pathogenesis of atherosclerosis seems to be endothelial injury. Based on earlier studies, which showed that the removal of endothelium in experimental hypercholesterolemic animals facilitated the development of the atherosclerotic plaque, the response to injury hypothesis was framed [29].

The multidirectional proatherogenic effects of inteleukin-1b, Il-6, TNF-α, and the fact that monocytes are the main type of inflammatory cells that infiltrate atherosclerotic plaques at an early stage of development indicate that this complex plays a different role in the stages of plaque development, atherosclerosis and its clinical consequences [30,31,32,33]. This issue prompted investigators to search for new biomarkers of plaque instability. Our results indicate significantly increased levels of these cytokines in patients with mixed hyperlipidemia and unstable atherosclerotic plaque. The reduction in TNF-α chemokine receptor expression, which is responsible for the influx of monocytes into atherosclerotic plaques, may be an important mechanism for the action of new lipid-lowering drugs with pleiotropic action, reducing monocyte diapedesis to atherosclerotic plaques [34].

Inflammation, along with activation of the coagulation pathway, is another important factor in the initiation and development of acute coronary syndrome (ACS). Local endothelial inflammation or systemic inflammation plays an important role in the formation and development of ACS. Consequently, various inflammatory markers were investigated by scientists in ACS-related research. For our study, we chose interleukin-6 and interleukin-18. IL-18 as the major pro-inflammatory cytokine may reflect the situation of the inflammatory response. Previous reports have shown that IL-18 may promote the progression of atherosclerosis, destabilize atherosclerotic plaque and accelerate plaque formation [35]. Youssef et al. showed that elevated plasma levels of IL-18 may be an important independent predictor of adverse clinical events within 30 days in ST-elevation myocardial infarction (STEMI) patients [36]. Upon rupture of the high risk atherosclerotic plaque in the coronary arteries, tissue factor (TF) initiates the extrinsic clotting pathway. Activating inflammation and clotting bring the system to a hypercoagulable state. In this cited study, TF and IL-18 levels were assessed in 67 ACS patients before and after angiography and/or PTCI [37]. The changing patterns of the coagulation status, inflammatory response and their correlation in patients with ACS were investigated to reveal the possible role of the inflammatory-coagulation network in the occurrence of ACS and provide clinical data for further research [36,37]. Similar results of increased levels of cytokines were obtained in our observation, which may indicate the involvement of the studied cytokines in the process of plaque rupture also in the carotid arteries, which, apart from ACS, may lead to other cardiovascular events, including stroke.

From the clinical point of view, it seems even more important that there are more and more studies proving the effectiveness of therapy in influencing interleukin concentrations and reducing cardiovascular risk. The first of such studies was the Canakinumab Anti-inflammatory Thrombosis Outcomes Study (CANTOS) study, which proved that the use of canakinumab (antibody against interleukin 1β) significantly reduces the occurrence of cardiovascular events, regardless of the concentration of circulating blood lipids [38]. Preliminary data from this experiment indicate that plasma Il-18 levels remain a determinant of residual risk, even after targeted interleukin-1β inhibition. Similarly, positive results have been reported by studies assessing the effect of tocilizumab (anti-IL-6 antibody) on reducing the ischemic area in patients with ST-elevation myocardial infarction [39].

Our study is not free of some limitations. The study did not assess clinical outcomes, such as the occurrence of cardiovascular events during prolonged follow-up. Additionally, the study population, although exceeding the required sample size, was relatively low. However, this is due to the fact that the number of patients treated with PCSK9 inhibitors in our country is rather low, and we chose very specific and difficult criteria to meet. Finally, our study excluded type 2 diabetic subjects and, therefore, the question of whether a similar beneficial effect on monocyte secretory function and systemic inflammation is also observed in diabetic patients requires further study.

## 5. Conclusions

Plasma levels of IL-6 and IL-18 were significantly higher in our group of patients with dyslipidemia and confirmed the presence of unstable atherosclerotic plaque compared to in the healthy control group. For this reason, these cytokines should be considered contemporary markers of the presence of unstable atherosclerotic plaques in the arteries. Therefore, in the future, biomarker panels and index scores will be useful and could be used in medicine to improve diagnosis and aid in prognosis. Additional studies on larger groups of patients are needed to unambiguously assess this relationship and are required to definitively assess the effect of the novel lipid-lowering therapy on the levels of atherosclerotic biomarkers and plaque rupture risk.

## Figures and Tables

**Table 1:** Baseline characteristics of patients (values are mean ± SD unless indicated otherwise; (##—*p* < 0.001 versus control; ns—not statistically significant).

	Control Group	Study Group
Number of patients	12	18
Age, years	47 ± 5	48 ± 6
BMI	28.1 ± 2.5	27.9 ± 2.4
Smokers, %	33.3	33.3
Systolic blood pressure, mmHg	130 ± 6	132 ± 5
Diastolic blood pressure, mmHg	82 ± 4	83 ± 4
Fasting glucose, mg/dL	92 ± 5	93 ± 6
Total cholesterol, mg/dL	163.4 ± 17.6	264.2 ± 38.5 ^##^
LDL cholesterol, mg/dL	93.5 ± 15.8	182.2 ± 32 ^##^
Non-HDL cholesterol, mg/dL	117.1 ± 20.6	221.2 ± 37 ^##^
HDL cholesterol, mg/dL	46.3 ± 5	43 ± 12.3 ^ns^
Triglicerydes, mg/dL	118.2 ± 27.5	192 ± 44.8 ^##^

**Table 2:** Correlation between cholesterols fraction levels and interleukins levels.

	Interleukin 18 Concentration	Interleukin 6 Concentration
Total cholesterol level	R = 0.71, *p* < 0.05	R = 0.52, *p* < 0.05
LDL cholesterol level	R = 0.66, *p* < 0.05	R = 0.45, *p* = 0.46
HDL cholesterol level	R = −0.28, *p* = 0.06	R = −0.04, *p* = 0.56
Non-HDL cholesterol level	R = 0.71, *p* < 0.05	R = 0.48, *p* < 0.05
Triglycerides level	R = 0.78, *p* < 0.01	R = 0.54, *p* < 0.05

## Data Availability

The data that support the findings of this study are available from the corresponding author (mbasiak@sum.edu.pl) on reasonable request.
